# Three controversies in health data science

**DOI:** 10.1007/s41060-018-0109-y

**Published:** 2018-03-07

**Authors:** Niels Peek, Pedro Pereira Rodrigues

**Affiliations:** 1 Division of Informatics, Imaging, and Data Science, School of Health Sciences, University of Manchester, Manchester, UK; 2 NIHR Greater Manchester Patient Safety Translational Research Centre, University of Manchester, Manchester, UK; 3 Centre for Health Technology and Services Research, Faculty of Medicine, University of Porto, Porto, Portugal

**Keywords:** Health data reuse, Health data analysis, Health data security

## Abstract

The routine operation of modern healthcare systems produces a wealth of data in electronic health records, administrative databases, clinical registries, and other clinical systems. It is widely acknowledged that there is great potential for utilising these routine data for health research to derive new knowledge about health, disease, and treatments. However, the reuse of routine healthcare data for research is not beyond debate. In this paper, we discuss three issues that have stirred considerable controversy among health data scientists. First, we discuss van der Lei’s 1st Law of Medical Informatics, which states that data shall be used only for the purpose for which they were collected. Then, we discuss to which extent routine data sources and innovations in analytical methods alleviate the need to conduct randomised clinical trials. Finally, we address questions of governance, privacy, and trust when routine health data are made available for research. While we don’t think that there is a definite “right answer” for any of these issues, we argue that data scientists should be aware of the arguments for different viewpoints, respect their validity, and contribute constructively to the debate. The three controversies discussed in this paper relate to core challenges for research with health data and define an essential research agenda for the health data science community.

## Introduction

The use of data and information in biomedicine and health has generated many controversies. Francis Bacon’s scientific method of experimental inductive reasoning divided Aristotelian and modern thinkers; John Snow’s application of mathematics and epidemiology to the cholera outbreak in London in 1854 was brilliant, but highly controversial at the time; and David Sackett’s pioneering work on evidence-based medicine in the last century was initially met with scepticism and mockery. Today, the application of novel data science methods to health data is stirring up new debates, about meaning, methodology, and morality.

The routine operation of modern healthcare systems produces a wealth of electronic data on a daily basis in electronic health records (EHRs), administrative databases, clinical registries, and other clinical systems. It is widely acknowledged that there is great potential for utilising these routine data for health research to derive new knowledge about health, disease, and treatments [30, 33, 38]. This could augment the knowledge physicians have acquired from their clinical experience, which involves the same patients but is less formal in its methodology and likely to be subject to bias [12]. However, there are concerns about the fitness of routine data for research; about the role that this new type of research should play within evidence-based medicine; and about the ethics of sharing data that was recorded in a private conversation with the doctor. In this paper, we discuss these three areas of debate, in each case providing different perspectives and arguments.

First, we approach van der Lei’s 1st Law of Medical Informatics, which states that data shall be used only for the purpose for which they were collected. Then, we discuss to which extent routine data sources and innovations in analytical methods have alleviated the need to conduct expensive randomised clinical trials. Data science enthusiasts claim that Big Data and machine learning can be used to answer all research questions, and traditionalists claim that there is no replacement for randomised experiments when we are interested in causation. Finally, we address access to data, as many researchers have the opinion that all medical and healthcare data should be made freely available to them without any restrictions, in order to accelerate research and improve medical knowledge, while data custodians fear privacy breaches and loss of public trust.

While we discuss these controversies within context of health research, they are not unique to the health domain and apply to many other areas of data science as well.

## Controversy I. Data shall be used only for the purpose for which they were collected

Each health system has as primary purpose to improve the health of its users. But as a side effect, health systems also produce data: about the health status of its users; when they came to see their doctor; which symptoms they reported; which diagnostic tests were conducted and what the results were; which treatments were given; and what the outcomes of those treatments were. Increasingly, these routine data are stored electronically rather than on paper, providing the opportunity to reuse them for other purposes, such as research and service improvement. In 1991, Johan van der Lei wrote that data shall be used only for the purpose for which they were collected and called this the 1st Law of Medical Informatics [44]. The law warns against secondary uses of healthcare data because, as van der Lei argued, health data can easily be misinterpreted outside the context where they were collected.

### Why data should only be used for the purpose for which they were collected

The 1st Law of Medical Informatics is supported by a vast list of known data quality issues that surround EHRs and other routine databases in health care [47]. Ancker et al. [2] have documented large variations in the way primary care physicians use EHRs. For instance, the annual average proportion of encounters with problem lists updated ranged from 5% to 60% per physician in their study. So, some physicians would rarely update the problem list, while others would do it in the majority of encounters with patients. This implies that problem lists, which are a key source of diagnostic information, cannot be interpreted without knowing the recording habits of the physicians who created them.

But the problems with secondary data range even widely, mostly related to a lack of importance given, by humans, to precision of recorded data. In 2009, Cruz-Correia and his colleagues reported on several anecdotal pieces of evidence of misinterpretations of secondary health data: diagnosis codes changing over time led to an erroneous assessment of increasing ischaemic myocardial infarction incidence; heterogeneity of and non-adherence to data collection protocols created a skewed assessment of flu diagnosis across an entire country; rounded timestamps and dates disabled the sound comparison of two emergency teams [13].

Next to physician-level sources of variation, there are system-level sources of bias in data that are routinely collected within healthcare systems [40]. Fundamentally, the presence of recorded data for a given individual depends on the individual having received services within that healthcare system. But there are often individuals who do not use healthcare services, because of barriers to access or by preference. Routine data are therefore an unreliable source to derive information about population health. Also, the validity of information provided by patients within clinical encounters is subject to systematic sources of reporting and recall bias inherent in any interview process [10], and clinicians may not detect and/or report relevant information. For instance, cardiovascular disease is often not recognised in women, even when they present with exactly the same symptoms as men [5]. Finally, patients may not adhere to diagnostic and treatment recommendations provided by their clinician, but adherence is rarely recorded.

One of the subtlest issues in the reuse of routine data for research is presence and missingness of information. Many studies that reuse routine data for research require that a visit or measurement took place within a specified time frame for patients to be included in the study. For instance, they may require that at least one blood pressure measurement was taken during the study period. It is widely assumed that this produces a sample that is representative of the population from which it is derived. However, this the imposition of just this one sufficiency requirement biases the sample towards sicker and older patients as was shown by Terris et al. [40]. Conversely, the presence of an observation (clinic visit) is itself meaningful and associated with poorer health outcomes—regardless of what was measured or observed during that visit. In a recent systematic review of 107 risk prediction studies that reused EHR data, no study assessed the role of such informative observations [18].

### Why data should also be used for health research

Physicians often struggle to apply medical knowledge to their patients because most evidence regarding the effectiveness of medical treatments has been generated through randomised clinical trials (RCTs) with highly selected populations under tightly controlled conditions. Also, most RCTs compare a given treatment against placebo but do not assess the comparative effectiveness of competing interventions. Data that are routinely collected in a healthcare system are, arguably, a much better source to inform treatment decisions, because these decisions are made in the very system that generated the data: the data describe the real-world population that is seen in every clinical practice, treated under the same uncontrolled conditions, and practice variation creates natural experiments between competing interventions from which we can learn which is most effective.

But perhaps the most compelling argument to reuse routine data for research is that this creates the opportunity to analyse very large data sets, with very long follow-up times, against very low cost. We can therefore use these data to answer questions that would never be answered with traditional studies such as RCTs. For example, Mathews and co-workers [31] performed a population-based study of diagnostic medical radiation exposure—a field that had thus far largely relied on information from a single study in Japanese atomic bomb survivors. They assessed the cancer risk following exposure to low-dose ionising radiation from diagnostic computed tomography (CT) scanning in a cohort of 10.9 million individuals in Australia, by linking EHRs from the Australian Medicare system to the Australian Cancer Database and the National Death Index. The mean follow-up time was 9.5 years for the group exposed to CT scanning and 17.3 years for the unexposed group. They found that cancer incidence was 24% greater in people exposed to CT scanning, after accounting for age, sex, and year of birth. This result allows clinical practitioners to much better weigh the diagnostic benefits of CT scans against their adverse effects.

Very large routine data sets cannot only be used to assess the safety and effectiveness of clinical procedures, but also to evaluate large-scale health policies—again something that is very hard without such data sets. During the 2000s, many Western countries introduced legislation to prohibit smoking in enclosed public places and the workplace. Yet while there was ample evidence that tobacco smoking is the primary cause of preventable mortality worldwide, little was known about the actual health benefits of such smoking bans. Been et al. [7] assessed the impact of UK smoke-free legislation, introduced in July 2007, on perinatal survival by linking individual-level data with death certificates for all registered singletons births in England over the time period 1995–2011, to obtain a data set of 52 thousand stillbirths and 10.2 million live-births. Using interrupted time series logistic regression analysis and counterfactual reasoning, it was estimated that in the first four years after the smoking ban, 991 stillbirths and 430 neonatal deaths were prevented. This result was only achievable through reuse of large-scale routine data.

## Controversy II. Big Data can never replace traditional medical research methods

The first RCT was conducted in 1948 [29], and it was soon recognised as the method of choice to assess safety and efficacy of pharmaceutical products. After the “mild sleeping pill” thalidomide caused severe birth defects and deaths in thousands in the early 1960s, legislation in most countries requires that RCTs are conducted to assess safety (first in animals and then humans) and efficacy before new drugs can enter the market. More generally, RCTs have become the cornerstone of evidence-based medicine and are broadly considered the only method that can provide unbiased estimates of causal effects. They are not only used to evaluate drugs but also medical devices; decision support tools; care bundles; patient self-management support; audit and feedback; and numerous other types of interventions that are applied within health care. The number of RCTs has grown exponentially over time, with currently 75 new trials being published every day [6].

Recently, prominent data scientists have come to the public with innovative analytical methods which can robustly use health data to derive strong statistical associations, promising safe adjustments to confounding factors. Along with the increasingly large amounts of routine data being made available every day, this has led to the corollary that RCTs are no longer needed to assess causal relationships: statistical associations can tell us “what works”—and that is all we need to know. However, this is certainly still not the opinion of traditional health researchers.

### Why Big Data can never replace traditional medical research methods

Research that reuses previously recorded health data has, by definition, a retrospective, observational (non-randomised) study design. This design is weak for assessing causal effects, especially when compared to prospective, randomised study designs. The main threat to validity is confounding, i.e. a lack of comparability between exposed and unexposed groups. The presence of confounding means that the exposed group is essentially different from the unexposed groups at baseline. Had the exposed actually been unexposed, their outcomes would still have been different from those in the actually unexposed group.

In both studies described in the previous section, we cannot exclude that the results are subject to confounding bias. For instance, it is conceivable that some of the people who underwent CT scanning in the study by Mathews et al. [31] were tested because they had unexplained symptoms that later turned out to be caused by a cancer diagnosis. The authors have minimised the risk that this has happened by excluding all patients that received a cancer diagnosis within one year after the CT scan—but we cannot exclude the possibility that there were other patients with a longer lead time. Similarly, neonatal care has probably improved during the years 2007–2011. Been et al.’s [7] interrupted time series design cannot distinguish the causal effects of such improvements from the effects of the smoking ban. Perhaps the number of stillbirths and neonatal deaths would have been much lower anyway, regardless of the smoking ban.

Bigger data sets are not going to solve this problem. Larger denominators can help to reduce variance—but not bias. They merely bring the risk that we fool ourselves more often because there will be more “statistically significant” results. Also, stratification of a population, aiming at homogeneous and well-described groups, leads to insufficient data because of the complexity of medical phenomena and the large number of potential confounders [45]. Similarly, more powerful analytical methods (e.g. structural causal modelling [35]) are not going to help us to escape from confounding bias, even if time is explicitly considered in the model [1], because there will always be unmeasured confounding in observational studies (Table 1). One could avoid that previously known confounders are not measured and try to identify previously unknown confounders by measuring a broad set of variables. However, there will always be a risk that previously unknown confounders remain unmeasured. And no analytical method, however powerful, can correct for something that wasn’t measured in the first place.

**Table 1:**
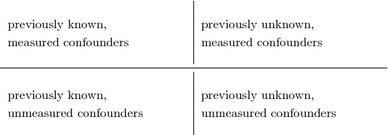
Four types of confounders

One could argue that in both examples discussed above, the benefits of having some causal effect estimate outweighs the disadvantage of a risk of bias. However, for most clinical interventions (such as drugs) it is perfectly feasible to conduct RCTs. With the health of so many patients at stake, it seems odd to take a risk if we can avoid it.

Apart from considerations about bias, there are further reasons why Big Data could not replace RCTs. It happens very often that one treatment is well established and an alternative treatment is newly developed. Typically, a lot of data will exist for the existing treatment but not for the new treatment, prohibiting a comparison through analysis of routine care data. Moreover, the clinical trial framework tries to minimise risks for individual patients. It is not possible to this without carefully designed controlled experiments. Uncontrolled experiments could lead to thousands of patients receiving suboptimal treatment and a high number of poor health outcomes (including deaths) which could have been avoided.

### Why Big Data should replace traditional medical research methods

Evidence-based medicine is a movement in crisis [19]. The pharmaceutical industry has been accused of overpowering trials to ensure that small differences will be statistically significant; setting trial inclusion criteria such that only those most likely to respond to treatment will participate; using surrogate outcome measures that have little to do with actual health; and selectively publishing positive studies [16]. Turner and colleagues showed that, from 74 FDA-registered trials of antidepressants, 23 were not published. From these trials, 22 (96%) had negative findings—while this was the case for only 14 out of 51 (27%) published trials [42].

There are more problems with RCTs. The US Department of Health and Human Services have estimated that the average clinical trial costs up to approval (phases I, II, and III) are $40m per drug. These costs have to be recovered through subsequent sales, but our societies are under pressure to spend less rather than more money on health care. Furthermore, RCTs typically have to exclude patients with co-morbidities. However, as the population ages and the prevalence of long-term conditions increases, it is becoming increasingly rare that patients with a single condition enter the consultation room. The evidence base that stems from RCTs does not apply to the real-world populations of today.

Routine data have the ability to provide evidence of safety and efficacy of medical interventions against a fraction of the costs of trials, and within representative populations and settings. Rather than having to set up an expensive trial design and execution apparatus, these studies can rely on extraction, linkage, and analysis of existing data. Setting up the technical and governance infrastructure to do this will require an initial investment (albeit much less than for the average RCT), but once this infrastructure exists, it can be reused many times against marginal extra costs: the UK Clinical Practice Research Datalink is an excellent example [24]. Because the costs are low, research using such data sources can be publically funded and avoid commercial biases. And there is no reason to exclude anyone, because the research is conducted retrospectively. Studies can evaluate representative cohorts that include complex patients with multi-morbidity and polypharmacy and that were treated in real-world settings.

Two types of data could then be used: wide (from large populations) and deep (a large amount of data per patient) [45]. Wide data might not be the best to support clinically relevant research at the patient level, due to lack of detail for the needed complexity of medical states and outcomes of each patient, and because these are mostly generated for administrative and reimbursement purposes, leading to problems of misinterpretation, as described above. Nonetheless, it may provide important insights for the exploration of clinical associations. On the other hand, deep data usually include temporal information on each patient, focusing on precise conditions at multiple scales, allowing for sound exploitation of clinical features.

An example of how routinely collected, real-world data can be used to assess the causal effects of treatment is provided by De Vries et al. [15]. They investigated the effect of cardiac rehabilitation on survival using a large Dutch insurance claims database (*n*=35,919). RCTs in this area are rare because it is commercially uninteresting, and the RCTs that have been conducted typically enrolled low-risk, middle-aged males without co-morbidities. In contrast, the study by De Vries et al. included all patients that were eligible to receive cardiac rehabilitation according to the Dutch clinical practice guidelines, including patients with common co-morbidities such as diabetes (20%), COPD/asthma (20%), and cancer (8%). Some well-known confounders of survival in this context (e.g. age, sex, diagnosis, and prior medical history) were present in the data but others, such as cardiac function, were missing. The authors solved this problem by using all available information, comprising hospital diagnoses–treatment combinations, outpatient prescriptions, medical devices, the occurrence of laboratory tests, GP visits, ICU days, and other services, to construct a large set of proxies for cardiac function and other potential confounders. Subsequently, generalised boosted regression [17] was used to estimate a propensity function, i.e. the probability of receiving cardiac rehabilitation as a function of 99 selected variables. These included variables such as “use of digitalis glycosides”, a potent cardiovascular drug that is typically prescribed for patients with poor cardiac function. The study showed that receiving cardiac rehabilitation led to a 35% reduction in mortality in this real-world population, a number which is much higher than what is typically found in trials [21].

Over the last two decades, tremendous improvements have been made in our understanding of the concept of causality, and in methods for causal inference from observational data [20, 22, 23]. Propensity scoring [3, 4] and instrumental variables [9, 14] are increasingly accepted as valid methods to address confounding in observational studies. In addition, innovative approaches to causal inference are studied in Machine Learning [28, 32, 41] and will soon make their way to applied health research to overcome limitations of existing methods.

## Controversy III. To protect the privacy of patients, health data should never be reused for research without explicit consent of the patients concerned

The cornerstone of medical informatics is data custody and curation for healthcare use. The cornerstone of data science is data analysis for hypothesis generation and validation. The cornerstone of health research is to reason upon health data for hypothesis confirmation and policy definition. Unfortunately, these three components of health data science present a conflicting approach to data access: while many researchers have the opinion that all medical and healthcare data should be made freely available to them without restrictions, data custodians fear privacy breaches and loss of public trust, especially with new restrictive European directives turned into law.

### Why health data should never be reused for research without explicit consent of the patients concerned

Patients must be able to trust doctors with their lives and health. An essential element of that trust relationship is the promise, made by every doctor, to treat patients as individuals and respect their dignity and their right to confidentiality. Without assurances about confidentiality, patients may be reluctant to seek medical help or to give doctors the information they need in order to provide good care.

Doctors are allowed to disclose personal information on their patients if this is justified by the public interest, and this creates the possibility to reuse routine patient data for research. However, it is not without risk to relevant values, and measures such as anonymisation do not solve all ethical and legal problems; people may, for example, have religious or moral objections to particular studies or concerns about stigma and breaches of privacy. In a pan-European survey among 20,882 citizens from 27 EU member countries, Patil et al. [34] found that most people are strongly averse to health insurance companies, private sector pharmaceutical companies, and academic researchers viewing their personal health data.

Aligned with this concern, the E.U. Data Protection Directive was adopted in 1995, enforcing the observance of three principles for personal data access: transparency, legitimate purpose, and proportionality. It has been recently translated into legislation, in April 2016, in an attempt to unify the application of such directive into national laws. The final approval included a particular nuance which might impact the entire world, since it defines that the regulation should also apply for all non-E.U. companies without any establishment in the E.U., provided that the processing of data is directed at E.U. residents; healthcare institutions are, clearly, not exempt.

If citizens’ preferences around health data sharing are ignored by their healthcare providers and governments, this can easily be met with large-scale public distrust. Trust in the protection of confidential health data in England reached an all-time low after the National Health Service (NHS) decided in December 2013 to establish a system for uploading and linking primary care EHR data for commissioning and research purposes, a programme known as “Care.data”. After a campaign by the British Medical Association, the Royal College of General Practitioners, and the privacy campaign group medConfidential that raised major public concern, the programme was suspended and eventually aborted. Ethicists have argued that the programme failed to respect the core values for a “social license for research”, i.e. participation is voluntary and governed by values of reciprocity, non-exploitation and service of the public good [11]. Any programme which attempts to make health data available for research should attempt to realise such a social licence and seek informed consent of the patients involved.

### Why health data should be made available for research without explicit consent of the patients concerned

While privacy campaign groups have often emphasised the risks of data sharing (e.g. privacy breaches and misuse of data), one could also argue that there is another side of the coin, i.e. risks due to the non-sharing of health data. The sharing of health data for research will increase our understanding of biology and medicine and thus lead to better decision-making and better health outcomes. This implies that non-sharing will, by necessity, lead to inferior decision-making and poor health outcomes, thus creating a moral imperative to share. Indeed, there have been devastating examples where non-sharing of data (e.g. non-publication of research findings) has been linked to harm to individuals. Also, some pharmaceutical companies have been accused to withhold data that was crucial to prevent detrimental effects of drugs [25]. Pharmacovigilance systems try to monitor the impact of drugs in the population, but the reporting of adverse drug events is still extremely underrated and highly dependent on health professionals’ and patients’ will and proactivity [36]. In general, though, there is a lack of evidence about the harms arising from non-sharing of information, and little indication of the scale of the problem.

A compelling argument in favour of the sharing of health data is the fact that many patients ask for it. For instance, www.usemydata.org is a movement for cancer patients, harnessing the patient voice to build confidence in the use of patient data for research. Patients that are cited on the website argue that it is patients’ responsibility to share data: “I think that, although it is the patient’s data and in the end it is their decision and their choice, but it is also their responsibility to make the data available for the benefit of others. Data has to be used responsibly and it has to be kept safe, but it has to be available for research” [43]. Also, the online patient community www.patientslikeme.org, which has more than 500,000 members, argues that health research is slowed down and the development of new treatments takes too long because most healthcare data is inaccessible.

An important question is whether routine healthcare data may be reused for research without consent—provided that proper information governance controls are in place to minimise the risk of reidentification, privacy breaches, or misuse of the data. This is allowed by law in most countries, and there are strong arguments for it. Obtaining consent of all the patients whose data are being used in such studies would be a laborious, expensive, and time-consuming operation without providing tangible benefits such as improved privacy. It would not possible for patients with serious mental illness, patients who are unconscious, and patients who have died. Moreover, opt-in consent would likely cause a strong selection bias.

Finally, if there are fewer barriers for researchers to access health data, then this will allow them to easier reproduce previous studies on other data sets. Concern about the reproducibility of scientific research has recently risen after reports that the results of biomedical experiments could not be replicated [8]. To maintain the integrity and trustworthiness of science, it is necessary to cultivate practices that facilitate reproducibility: wieldy access to health data is one of them.

## Discussion

We have presented three controversies in health data science that tend to ignite considerable debate whenever, and wherever, they are brought to the table. During the European Association for Data Science (EuADS) inaugural conference that was held in Luxembourg, 7–8 November 2016, the three controversies were presented, and delegates were invited to give their opinions on each of them through an online voting website [39] and by exchanging arguments in favour and against them. Table 2 presents the results from the voting. It shows that, even among a homogenous group of data scientists, there is no consensus about these three issues. There was a lively debate around each of them.

**Table 2:** Results from polling during the European association for data science inaugural conference, Luxembourg, 7–8 November 2016

	Agree (%)	Do not agree (%)
Data shall be used only for the purpose for which they were collected	27	73
Big Data can never replace traditional medical research methods	76	24
To protect the privacy of patients, health data should never be reused for research without explicit consent of the patients concerned	63	37

While we have presented these controversies as “yes or no” questions, we don’t think that choosing sides is right solution for any of them. For each of the three controversies, there are very good arguments both in favour and against them—as we have explained in the preceding sections. Rather, data scientists should be aware of these arguments, respect their validity, and contribute constructively to the debate. In our opinion, the three controversies that are discussed in this paper should be part of every data science curriculum.

Although a systematic review of the literature would possibly better highlight all the controversies surrounding health data science, we have decided to focus on those three that, from our experience and personal debates with the community, create higher impact in the conclusions drawn from current methods for health data science research, as we believe both methodologies would result in a quite similar message to the scientific community. We also acknowledge that the three controversies discussed in this paper are not unique to the health domain and apply to many other domains as well. However, it was beyond the scope of our work to rigorously review the broader literature on these topics.

The three controversies here discussed lay in the heart of the three sources of evidence for evidence-based medicine, as Sackett would put it [37], which include the personal experience of the clinician (stored in medical records); the published evidence from quality research (produced according to traditional or innovative study designs); and the values and needs of the individual patient (strengthen by the privacy vs accessibility concerns of sensitive data). Therefore, they clarify that the discipline of Health Data Science is, in fact, a nonlinear process connecting health informatics, data science, and clinical and health services research.

Perhaps even more important is the fact that these controversies relate to core challenges for research with data: they provide generalizable insights and define an essential research agenda for the health data science community. The first controversy (“Data shall be used only for the purpose for which they were collected”) teaches us that data cannot be interpreted without carefully considering the context in which they where gathered. Therefore, we need better tools to capture, represent, and utilise context information—for instance in the form of meta-data that describe how data were captured; when; by whom; and for what purpose. Analytical methods should use these meta-data to avoid incorrect interpretations and biases. The second controversy (“Big Data can never replace traditional medical research methods”) reflects that causation is at the core of human reasoning but peripheral to statistical inference—and this will always create a tension. Ignoring the importance of causality is not the solution, but it would be equally ignorant to deny that the Big Data era has created promising, new opportunities for knowledge discovery from data. The decision whether an RCT should be conducted to evaluate a new intervention should be based on trading off risk and costs. For low-risk, low-cost interventions such as cardiac rehabilitation, real-world evidence from retrospective cohort studies should be acceptable, while for other interventions evidence from RCT is required. The third controversy (“To protect the privacy of patients, health data should never be reused for research without explicit consent of the patients concerned”), finally, relates to an issue that is perhaps most fundamental to all data science. We live in a world in which our ability to capture personal data far exceeds our understanding of how to manage issues of trust, privacy, and consent. This has far-reaching consequences for both individuals and society. Data scientists should create the tools to help individuals to protect their privacy, empower them to have control over what happens to their personal data, and, at the same time, maximise the benefits of data for society. This is especially crucial in the health domain where both the privacy risks for individuals and potential benefits to society are higher than in other domains. New models of data sharing (for instance through data safe havens [26]) and innovative, privacy-preserving analytical methods [27, 46] are promising avenues of research that can make this happen.
